# A critical role for STIM1 in filopodial calcium entry and axon guidance

**DOI:** 10.1186/1756-6606-6-51

**Published:** 2013-12-01

**Authors:** Sangwoo Shim, James Q Zheng, Guo-li Ming

**Affiliations:** 1 Departments of Cell Biology and Neurology, Emory University School of Medicine, Atlanta, GA 30078, USA; 2 Center for Neurodegenerative Diseases, Emory University School of Medicine, Atlanta, GA 30322, USA; 3 Institute for Cell Engineering, Johns Hopkins University School of Medicine, Baltimore, MD 21205, USA; 4 Department of Neurology, Johns Hopkins University School of Medicine, Baltimore, MD 21205, USA; 5 The Solomon H. Snyder Department of Neuroscience, Johns Hopkins University School of Medicine, Baltimore, MD 21205, USA

**Keywords:** Axon guidance, STIM1, SOCE, TRPC1, Calcium, Netrin-1, Filopodial Ca^2+^ entry, Ca^2+^ oscillation, Calcium homeostasis

## Abstract

**Background:**

Stromal interaction molecule 1 (STIM1), a Ca^2+^ sensor in the endoplasmic reticulum, regulates store-operated Ca^2+^ entry (SOCE) that is essential for Ca^2+^ homeostasis in many types of cells. However, if and how STIM1 and SOCE function in nerve growth cones during axon guidance remains to be elucidated.

**Results:**

We report that STIM1 and transient receptor potential channel 1 (TRPC1)-dependent SOCE operates in *Xenopus* spinal growth cones to regulate Ca^2+^ signaling and guidance responses. We found that STIM1 works together with TRPC1 to mediate SOCE within growth cones and filopodia. In particular, STIM1/TRPC1-dependent SOCE was found to mediate oscillatory filopodial Ca^2+^ transients in the growth cone. Disruption of STIM1 function abolished filopodial Ca^2+^ transients and impaired Ca^2+^-dependent attractive responses of *Xenopus* growth cones to netrin-1. Finally, interference with STIM1 function was found to disrupt midline axon guidance of commissural interneurons in the developing *Xenopus* spinal cord in vivo.

**Conclusions:**

Our data demonstrate that STIM1/TRPC1-dependent SOCE plays an essential role in generating spatiotemporal Ca^2+^ signals that mediate guidance responses of nerve growth cones.

## Background

Guided axonal growth and regeneration depend on the motile growth cone at the tip of axons to extend and navigate through a complex environment to reach specific targets for neuronal connections. It is well established that the nerve growth cone needs to maintain an optimal range of intracellular Ca^2+^ concentration ([Ca^2+^]_i_) for its motility and responses to extracellular cues [1]. The cytoplasmic Ca^2+^ homeostasis is regulated by Ca^2+^ entry from the extracellular environment, internal Ca^2+^ release and replenishment of the intracellular store [2,3]. However, how neuronal growth cones coordinate guidance cue-induced Ca^2+^ influx, internal Ca^2+^ release and Ca^2+^ store replenishment to maintain proper guidance behaviors is unknown. Store-operated Ca^2+^ entry (SOCE) was originally characterized in non-excitable cells as an indispensable Ca^2+^ influx mechanism to replenish internal stores [2,3]. It is triggered by Ca^2+^ depletion from ER through the ER Ca^2+^ sensor protein, stromal interacting molecule 1 (STIM1). In response to Ca^2+^ depletion, STIM1 oligomerizes and translocates to ER and plasma membrane junctions, where it interacts with and activates store-operated Ca^2+^ (SOC) channels that include TRPC1 and Orai1 proteins [2,3].

In the nervous system, SOCE has been seen to exist in a number of cell types [4-7] and implicated in synaptic plasticity, axon branching, neuropathic pain and fly motor circuit function [6-10]. However, the existence of SOCE and STIM1, and their potential contribution to the intracellular Ca^2+^ homeostasis and signaling in axon guidance is not well established. Axonal growth cones are highlighted by two types of actin-based motile membrane protrusions, filopodia and lamellipodia [11]. Of these two structures, lamellipodia are considered to be responsible for growth cone locomotion, whereas filopodia are believed to function in sensing of the environment during axon pathfinding [11-13]. Interestingly, rapid Ca^2+^ transients in growth cone filopodia have been shown to be involved in growth cone responses to extracellular cues [14,15]. But how Ca^2+^ signals are generated in filopodia and whether SOCE is involved in this process remain unknown. Here we report that SOCE operates in *Xenopus* spinal growth cones and depends on STIM1 and TRPC1. Importantly, we find that SOCE mediates spontaneous and netrin-1-potentiated filopodial Ca^2+^ entries within growth cones. We further provide evidence that STIM1- and TRPC1-dependent SOCE is required for attractive guidance responses of growth cones to netrin-1. Finally, we show that STIM1 is required for midline axon guidance of commissural interneurons in the developing *Xenopus* spinal cord *in vivo*. Our data suggest that SOCE is an essential component of intracellular Ca^2+^ homeostasis and signaling that regulate neuronal growth cone guidance.

## Results

### Cloning and expression of Xenopus STIM1

**Figure 1:**
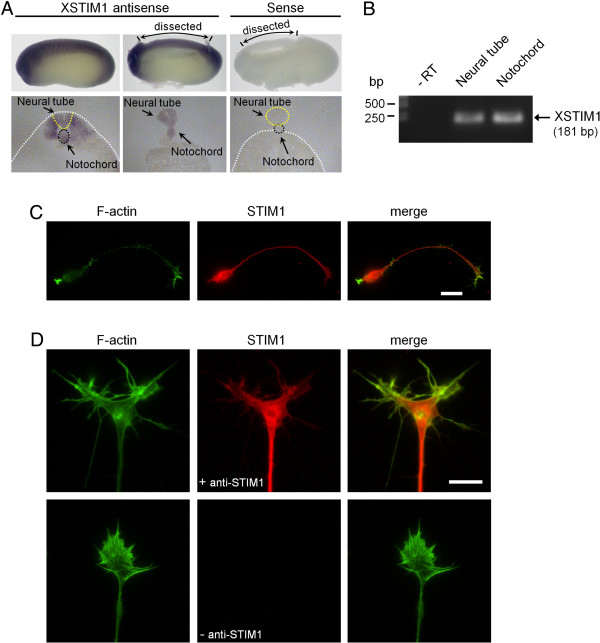
***Xenopus *STIM1 is expressed in developing neural tissues and neuronal growth cones. (A)** Sample images of whole-mount (top) and cross-section (bottom) *in situ* hybridization analysis of the mRNA expression of XSTIM1 in developing *Xenopus* embryos. Left, antisense; right, sense probe. Dotted lines delineate the boundaries of neural tube and notochord. **(B)** RT-PCR detection of XSTIM1 mRNA from RNA samples extracted from state 25-26 *Xenopus* neural tube and notochord tissues. –RT lane is the negative control of the RT-PCR on neural tube tissue RNA in the absence of a reverese transcriptase. **(C)** Representative immunofluorescence images of cultured *Xenopus* spinal neurons labeled for STIM1 (red) and F-actin (phalloidin: green). Scale bar: 20 μm. **(D)** Representative immunofluorescence images of growth cones labeled for STIM1 (red) and F-actin (green). Negative control processed without STIM1 antibody (without STIM1, bottom) shows absence of immunolabeling. Scale bar: 10 μm.

We first cloned *Xenopus* STIM1 (XSTIM1; 668 a.a.), which exhibited 72.8% identity to human STIM1 (685 a.a.; Additional file 1: Figure S1). Whole-mount *in situ* hybridization of developing *Xenopus* embryos showed that STIM1 is highly expressed in the dorsal part of the developing embryo, including the neural tube at stages when active axon guidance occurs (Figure 1A, top panels). Coronal sections of *Xenopus* embryos confirmed the expression of STIM1 mRNA in the neural tube, as well as in the notochord and somites (Figure 1A, bottom panels). RT-PCR analysis from dissected tissues further confirmed that XSTIM1 mRNA is expressed in neural tube and notochord of early tailbud *Xenopus* embryos (Figure 1B). Immunofluorescence analysis using anti-STIM1 antibody and fluorescent phalloidin to stain F-actin showed that STIM1 protein is ubiquitously distributed in *Xenopus* spinal neuron, including soma, neurites and growth cones (Figure 1C). Robust expression of XSTIM1 throughout the growth cone and in filopodia is better seen at higher magnifications (Figure 1D). These results show that *Xenopus* STIM1 is expressed in developing neural tissues and neuronal growth cones of developing axons.

### STIM1- and TRPC1-dependent SOCE in Xenopus neuronal growth cones

**Figure 2:**
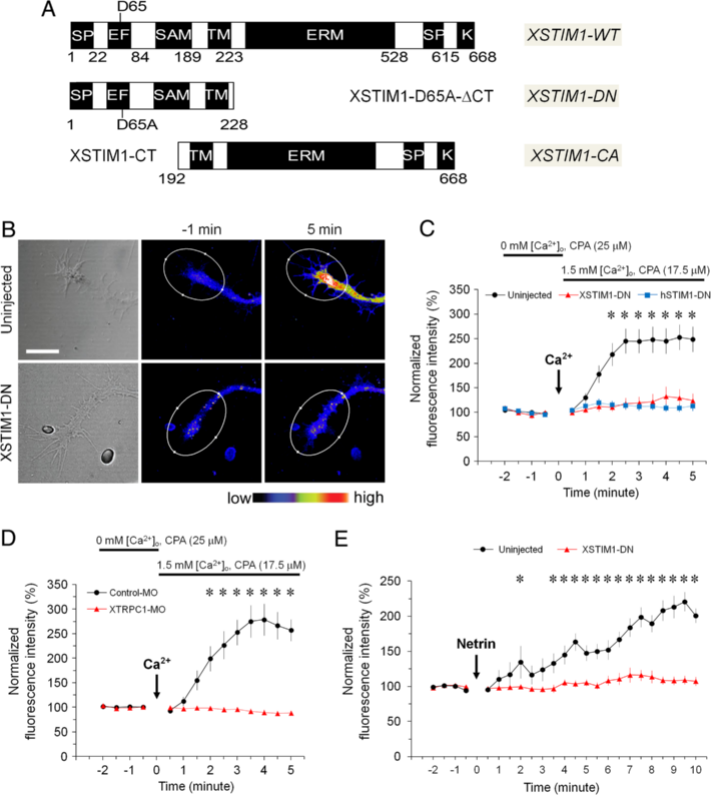
**STIM1-dependent SOCE operates and mediates netrin-1-induced Ca**^**2+ **^**elevation in *Xenopus *neuronal growth cones. (A)** A schematic diagram of full-length wild-type (WT) and mutant constructs of XSTIM1. **(B)** Bright field and pseudocolor images of fluo-4 fluorescence of growth cones of *Xenopus* spinal neurons from the uninjected or mcherry-XSTIM1-DN injected embryos in the presence of CPA in Ca^2+^-free media, before and after the re-addition of 1.5 mM Ca^2+^ bath solution. Pseudocolors indicate Ca^2+^ levels, with white as the highest and black as the lowest. Scale bar: 10 μm. **(C)** Summary of internal Ca^2+^ store depletion-induced Ca^2+^ entry in growth cones at different time points before and after re-addition of 1.5 mM Ca^2+^. The fluorescence intensity was normalized to the average fluorescence intensity of 2 min baseline levels prior to Ca^2+^ re-addition. Values represent mean ± s.e.m. (n = 25 for control, n = 10 for XSTIM1-DN and n = 19 for hSTIM1-DN; * indicates *P* < 0.01; Bootstrap-test). **(D)** XTRPC1 is required for store depletion-evoked Ca^2+^ entry in neuronal growth cones. Summary of internal Ca^2+^ store depletion-induced Ca^2+^ entry in growth cones from the control-MO or XTRPC1-MO injected embryos at different time points before and after the re-addition of 1.5 mM Ca^2+^. Values represent mean ± s.e.m. (n = 12 for control, and n = 13 for XTRPC1-MO; * indicates *P* < 0.01; Bootstrap-test). **(E)** XSTIM1 is required for netrin-1-induced Ca^2+^ elevation in growth cone. Summary of time course of Ca^2+^ changes in neuronal growth cones from uninjected or mCherry-XSTIM1-DN mRNA injected embryos. The fluorescence intensity was normalized to the average fluorescence intensity of 2 min baseline levels prior to the netrin-1 application (10 ng/ml). Values represent mean ± s.e.m. (n = 6 for control and n = 9 for XSTIM1-DN; * indicates P < 0.05; Bootstrap-test).

To study the function of STIM1 in growth cones, we generated wild-type (XSTIM1-WT) and two mutant forms of XSTIM1: a dominant negative form (DN, XSTIM1-D65A-ΔCT) and a constitutively active form (CA, XSTIM1-CT) (Figure 2A) based on previous studies of mammalian STIM1 mutants [16]. We first examined whether STIM1-dependent SOCE operates in neuronal growth cones by Ca^2+^ imaging analysis using fluo-4 calcium indicator. *Xenopus* neurons were first bathed in Ca^2+^-free media with cyclopiazonic acid (CPA; 25 μM), an endoplasmic reticulum Ca^2+^ pump inhibitor, to deplete intracellular Ca^2+^ stores. Subsequent addition of extracellular Ca^2+^ induced a marked and rapid rise in the intracellular Ca^2+^ concentration ([Ca^2+^]_i_) in the growth cone (Figure 2B and C), suggesting the presence of functional SOCE in neuronal growth cones [4-7]. Importantly, we found that the SOCE was largely abolished in growth cones expressing either dominant negative *Xenopus* STIM1 (XSTIM1-DN, Figure 2B and 2C) or dominant negative human STIM1 (hSTIM1-DN) (Figure 2C), indicating that the elevation in [Ca^2+^]_i_ requires the function of STIM1. In addition, knock-down of TRPC1 channels by morpholino oligonucleotides against XTRPC1 (XTRPC1-MO) abolished SOCE (Figure 2D). Therefore, TRPC1 is also an essential component of store-operated Ca^2+^ (SOC) channel complex as suggested by previous studies in saliva gland cells [17]. These results show that store-operated Ca^2+^ entry operates in neuronal growth cones and requires STIM1 and TRPC1.

### STIM1-dependent SOCE mediates netrin-1-induced Ca^2+^ elevation in growth cones

Netrin-1 is known to guide axonal growth cone through a Ca^2+^-dependent pathway [1,18] and netrin-1-induced increase in [Ca^2+^]_i_ in growth cones requires both intracellular Ca^2+^ release and Ca^2+^ influx through channels on the plasma membrane [19-21]. Consistent with previous studies [21-23], bath application of netrin-1 (10 ng/ml final concentration) induced a significant rise in [Ca^2+^]_i_ in *Xenopus* growth cones (Figure 2E). Importantly, overexpression of XSTIM1-DN abolished the sustained Ca^2+^ elevation within neuronal growth cones in response to netrin-1 (Figure 2E). Previous studies from ours and others have shown that TRPC1 is required for netrin-1-induced sustained Ca^2+^ elevation [21,23]. We have observed an association of TRPC1 with STIM1 in *Xenopus* brain lysates (Additional file 2: Figure S2), similar to what has been shown in mammalian cells [2,16,24]. Therefore, netrin-1 may activate STIM1-dependent SOCE through TRPC1 in neuronal growth cones.

### STIM1-dependent SOCE generates filopodial Ca^2+^ entries in Xenopus neuronal growth cones

**Figure 3:**
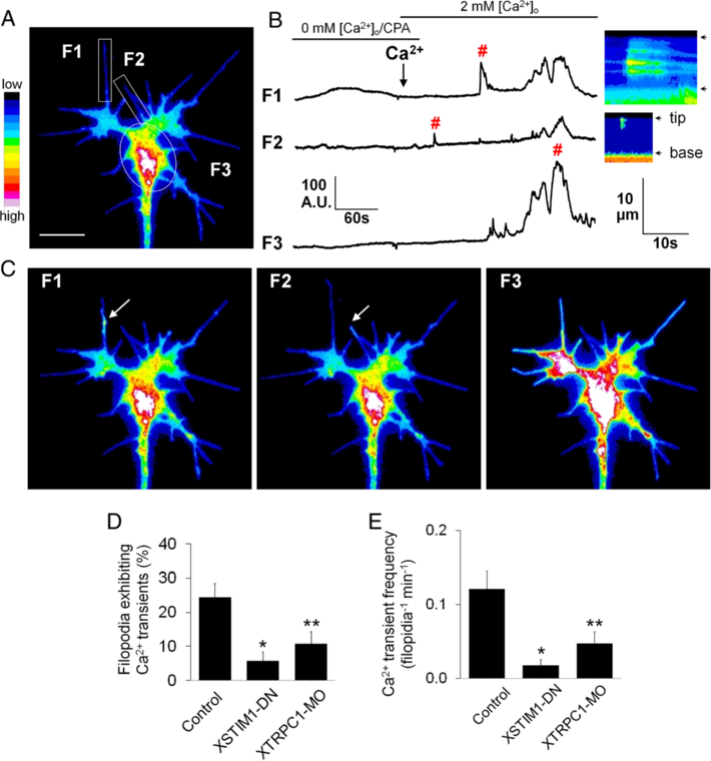
**STIM1-dependent SOCE generates filopodial Ca**^**2+ **^**entries in *Xenopus *neuronal growth cones. (A)** A pseudocolored Lck-GCaMP3 fluorescent Ca^2+^ image of growth cone showing rectangular ROIs (region of interest) used to measure fluorescent intensities over time. Pseudocolors indicate Ca^2+^ levels, with white as the highest and black as the lowest. Scale bar, 10 μm. **(B)** Representative traces of Lck-GCaMP3 fluorescent Ca^2+^ signals profile measured in two filopodia (F1, F2) and a growth center (F3) over 7 min period of store-depletion and re-addition of extracellular Ca^2+^. Images were captured at 200 milliseconds intervals. #, indicates filopodial and global Ca^2+^ transients that are shown in **(C)**. Right images are kymographs generated from a segmented line along the filopodia from the tip to the base using NIH ImageJ. The arrowheads denote tip and base of filopodia. **(C)** Representative pseudocolored Lck-GCaMP3 fluorescent Ca^2+^ images at the time point as indicated by # in *B*. The arrows show the initiation of filopodial Ca^2+^ transients. **(D-E)** The incidence **(D)** and frequency **(E)** of filopodial Ca^2+^ transients were determined in control (n = 21), XSTIM1-DN (n = 12), XTRPC1-MO (n = 10) expressing filopodia. *P < 0.005 and **p < 0.05 compared with control condition using t-test. Values represent mean ± s.e.m.

Localized Ca^2+^ transients in filopodia play an important role in filopodial motility and growth cone responses to extracellular cues [14]. We thus examined the potential role for SOCE in Ca^2+^ entry in growth cone filopodia. Here we used a membrane tethered calcium indicator Lck-GCaMP3 [25] to reliably monitor Ca^2+^ influx through the plasma membrane. Using a similar protocol as in Figure 2B-D, we measured both the incidence and frequency of fast Ca^2+^ entries in filopodia by high-speed (30 frames/sec) wide-field fluorescent imaging. We found that store Ca^2+^ depletion and re-addition of external Ca^2+^ experiment led to Ca^2+^ entries in about 25% filopodia (Figure 3; Additional file 3: Movie 1). High frequency time-lapse traces of the integrated intensity of the Lck-GCaMP3 fluorescence and a kymograph representation clearly demonstrated that filopodial Ca^2+^ entries are independent of growth cone Ca^2+^ transients (Figure 3A-C; Additional file 3: Movie 1). Importantly, both the incidence and frequency of these filopodial Ca^2+^ entries were largely abolished by inhibition of XSTIM1 with XSTIM1-DN overexpression (Figure 3D and E; Additional file 4: Movie 2) or TRPC1 knockdown by XTRPC1-MO (Figure 3D and E; Additional file 5: Movie 3). These results thus indicate that STIM1- and TRPC1-dependent SOCE is indispensable for filopodial Ca^2+^ entries within neuronal growth cones.

**Figure 4:**
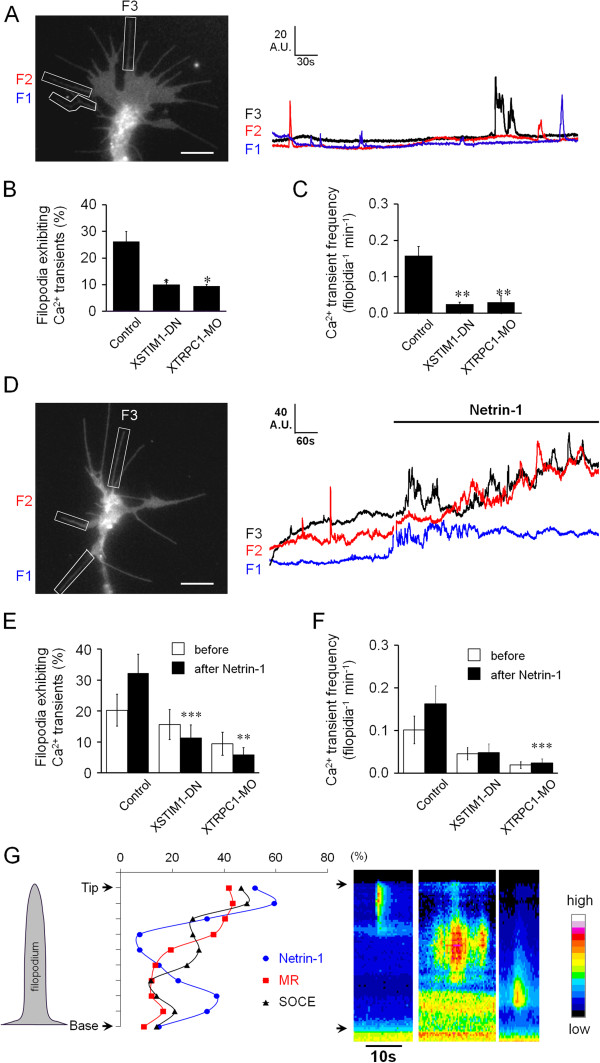
**STIM1/TRPC1-dependent SOCE mediates the spontaneous and netrin-1-potentiated filopodial Ca**^**2+ **^**entries. (A)** Left panel; a Lck-GCaMP3 fluorescent Ca^2+^ image of a *Xenopus* spinal growth cone showing three ROIs (F1, F2 and F3) encompassing the filopodia used to measure fluorescent intensities over time (right panels). Scale bar, 10 μm. The incidence **(B)** and the frequency **(C)** of the spontaneous filopodial Ca^2+^ transients were significantly attenuated by XSTIM1-DN (n = 30) or XTRPC1-MO (n = 30), when compared to the control (n=34; *P < 0.001 and **p < 0.005, Student’s t-test). Values represent mean ± s.e.m. **(D)** A Lck-GCaMP3 fluorescent Ca^2+^ image of a growth cone showing three ROIs (left panel) and their representative traces of Ca^2+^ signals in three filopodia (F1, F2, F3) before and after bath application of netrin-1 (10 ng/ml) (right panel) in the presence of Sp-cAMP (25 μM). Scale bar, 10 μm. **(E-F)** Netrin-1 potentiated the incidence **(E)** and the frequency **(F)** of filopodial Ca^2+^ transients in spinal growth cones (control; n = 14) and this potentiation was abolished by XSTIM1-DN (n = 8) and XTRPC1-MO (n = 10). **P < 0.005 and ***p < 0.05 (Student’s *t*-test). Values represent mean ± s.e.m. **(G)** Filopodia tips are the major site of initiation of filopodial Ca^2+^ entry as revealed by kymographs of Ca^2+^ signals in filopodia using Lck-GCaMP3 in modified Ringers saline (MR; n = 67), netrin-1 exposure (n = 27) and Ca^2+^ re-addition after depletion (SOCE; n = 43). The y axis represents the path distance along the filopodia divided into 10 portions and the x axis represents time. The arrows denote the tip and base of filopodia.

Previous studies have revealed that *Xenopus* growth cones exhibit spontaneous filopodial Ca^2+^ transients that are closely associated with growth cone motility [14,15,26]. Using Lck-GCaMP3, we also observed robust periodic, spontaneous calcium entries in filopodia of *Xenopus* growth cones in a Modified Ringer solution that contains 1 mM extracellular Ca^2+^ (Figure 4A; Additional file 6: Movie 4). Importantly, both the incidence and frequency of the entries were also substantially reduced by inhibition of STIM1 and TRPC1 function (Figure 4B and C; Additional file 7: Movie 5 and Additional file 8: Movie 6), suggesting that STIM1/TRPC1-dependent SOCE is involved in generating and maintaining oscillatory patterns of spontaneous filopodial Ca^2+^ transients.

Bath application of netrin-1 (10 ng/ml final concentration) was found to potentiate both the incidence and frequency of filopodial Ca^2+^ entries of *Xenopus* spinal growth cones (Figure 4D-F; Additional file 9: Movie 7), consistent with previous study using Fluo-4 [15]. We found that this increase in filopodial Ca^2+^ entries by netrin-1 was abolished when STIM1 function was inhibited by XSTIM1-DN (Figure 4E and F). Over-expression of morpholino against XTRPC1 (XTRPC1-MO) also compromised the potentiation of filopodial Ca^2+^ entries by netrin-1 (Figure 4E and F). Therefore, STIM1/TRPC1-dependent SOCE mediates the netrin-1-dependent potentiation of oscillatory filopodial Ca^2+^ entries in neuronal growth cones.

The membrane tethered calcium indicator lck-GCaMP3 also provides an opportunity to map the entry sites of Ca^2+^ in filopodia. We thus analyzed the sites of initial filopodial Ca^2+^ entry in *Xenopus* growth cones by kymography analysis. We found that, although the initial sites of Ca^2+^ entry distributed throughout the length of a filopodium, a large portion of the Ca^2+^ entry sites (42-59%) were found at the filopodial tip (Figure 4G). When filopodial Ca^2+^ entries under different conditions (store-operated, spontaneous, and netrin-1-induced) were examined, no difference was seen on the location of Ca^2+^ entry sites in filopodia (Figure 4G). Therefore, the tip of the filopodia appears to be the primary site of SOCE-mediated Ca^2+^ entry in nerve growth cones.

**Figure 5:**
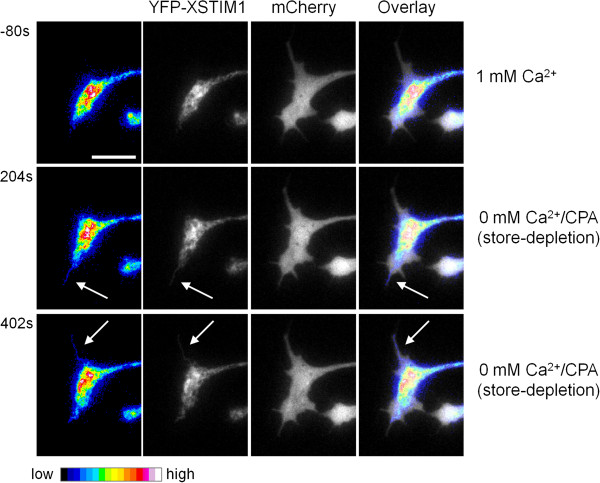
**Dynamic translocation of STIM1 into neuronal filopodia in response to store-depletion.** Representative time-lapse fluorescent images of growth cone expressing YFP-XSTIM1 before (1 mM Ca^2+^) and after store Ca^2+^ depletion (0 mM Ca^2+^/CPA). mCherry was co-expressed to mark filopodia and growth cone. Images were pseudocolored to enhance the observation of intensity changes. The arrows indicate newly translocated XSTIM1 proteins into neuronal filopodia. Scale bars: 10 μm.

STIM1 proteins reside predominantly in the ER, and undergo rapid and reversible translocation into ER-plasma membrane junctions to interact with and activate SOC channels following store depletion in non-excitable cells [27]. Live cell imaging of *Xenopus* neurons expressing YFP tagged STIM1 showed that YFP-XSTIM1-WT appeared to translocate into filopodia after store Ca^2+^ depletion, as revealed by pseudocolored images and phase overlay images with mCherry (Figure 5; Additional file 10: Movie 8). Together with the presence of STIM1 in *Xenopus* growth cones and their filopodia by immunostaining (Figure 1D), these results suggest an intriguing possibility that STIM1 proteins may become spatially reorganized into growth cone filopodia after activation by store-depletion to further activate SOC channels that may include TRPC1.

### XSTIM1 is required for growth cone guidance

**Figure 6:**
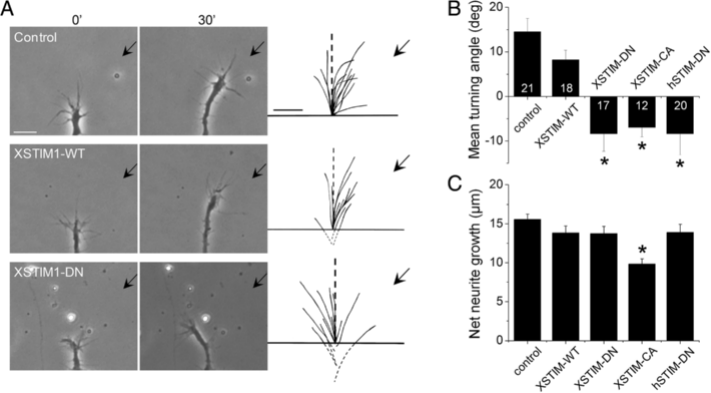
**XSTIM1 is required for attractive turning responses of neuronal growth cones to a netrin-1 gradient. (A)** Sample images of growth cone turning responses in a gradient of netrin-1 of a control *Xenopus* spinal neuron, and neurons derived from embryos injected with mRNA encoding wild-type (YFP-XSTIM1-WT), or dominant negative mutant (YFP-XSTIM1-DN). The left two columns of images show neuronal growth cones at the start (0 min) and the end of exposure (30 min) to a netrin-1 gradient (5 μg/ml in the pipette). The right column shows superimposed trajectories of neurite extension during the 30' period for a sample population of 12 neurons under the each condition. The origin is the center of the growth cone and the original direction of growth is vertical. Arrows indicate the direction of the gradient. Scale bars: 10 μm. **(B)** Summary of mean turning angles of growth cones in responses to a gradient of netrin-1 under different conditions. The number associated with the bar graph indicates the number of growth cones analyzed. Values represent mean ± s.e.m. (* indicates *P* < 0.01; Bootstrap-test). **(C)** Summary of net neurite growth during the 30 minutes turning assay under different conditions. Values represent mean ± s.e.m. (* indicates *P* < 0.05; Bootstrap-test).

To test whether STIM1-dependent SOCE is required for growth cone guidance in response to netrin-1, we employed a well-established *in vitro* growth cone turning assay [19,20,23,28]. Previous studies have shown that netrin-1, a classical guidance cue, induces growth cone turning responses that are mediated by Ca^2+^ from both extracellular and intracellular sources [19-21,29]. In a microscopic gradient of netrin-1 (5 μg/ml in the pipette, ~5 ng/ml reaching the growth cone), *Xenopus* growth cones of overnight culture (12-20 hrs) without laminin coating exhibited robust chemoattractive turning within 30 minutes (Figure 6A). Importantly, expression of XSTIM1-DN or XSTIM1-CA in *Xenopus* spinal neurons completely abolished netrin-1-induced attraction, and interestingly resulted in repulsion (Figure 6A and B). Expression of wild-type STIM1 (WT) produced no effect on netrin-1-induced attractive turning (Figure 6A and B). Overexpression of the dominant negative human STIM1 (hSTIM1-DN) [16] also eliminated netrin-1-induced attraction and converted it to repulsion (Figure 6B). The neurite extension rate in a netrin-1 gradient was not significantly different under these conditions [29], except XSTIM1-CA which slightly reduced the growth rate (Figure 6C). Thus, proper function of XSTIM1 is essential for netrin-1-induced growth cone turning responses of *Xenopus* spinal neurons *in vitro*. Together with the previous studies showing that TRPC1 knockdown abolished the netrin-1-induced attractive growth cone turning responses [20,21], the results indicate that STIM1/TRPC1-dependent SOCE may play a critical role in growth cone guidance.

**Figure 7:**
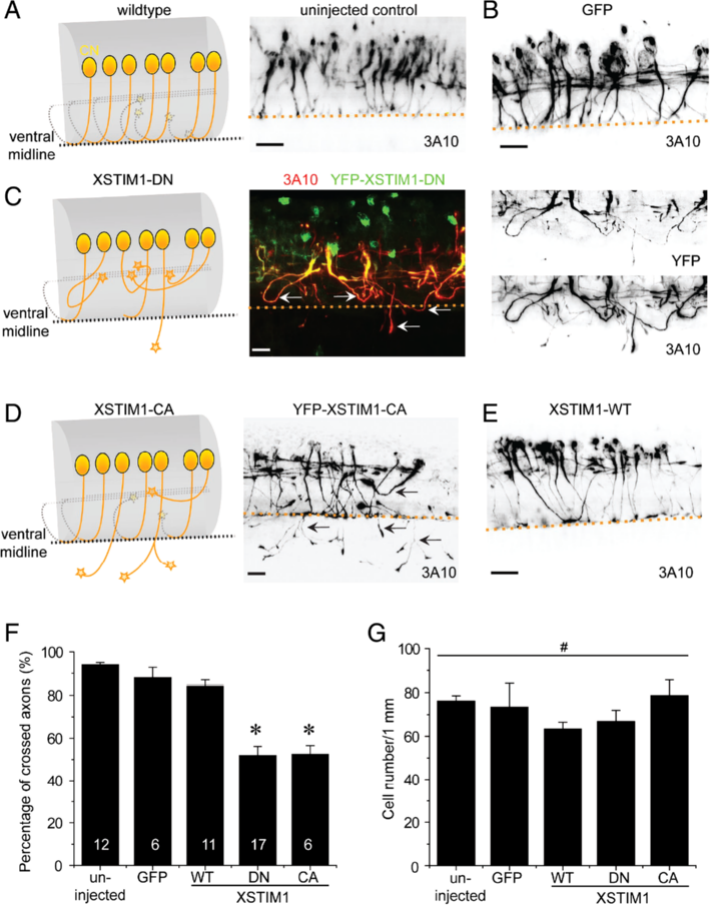
**XSTIM1 is required for midline axon guidance of commissural interneurons in the developing *Xenopus *spinal cord. (A-E)** Sample images of the sagittal view of commissural interneurons and their axonal projections in the *Xenopus* spinal cord from stage 25-26 embryos. Shown are schematic diagrams and confocal z-stack projection images of 3A10 staining of commissural interneuron axons from uninjected embryos, or embryos injected with mRNAs for GFP **(B)**, YFP-XSTIM1-DN **(C)**, YFP-XSTIM1-CA **(D)**, or YFP-XSTIM1-WT **(E)**, at the two to four cell-stage to manipulate a sub-population of neurons. Dotted line represents ventral midline; arrows point to mis-targeted axons. Scale bars: 20 μm. **(F)** Quantification of the percentage of 3A10^+^ commissural interneurons with normal midline crossing under different experimental conditions. The number associated with the bar graph indicates the number of embryos examined under each condition. Values represent mean ± s.e.m. (* indicates *P* < 0.01; Bootstrap-test). **(G)** Summary of the density of 3A10^+^ commissural neurons under each condition. The same embryos as in **(F)** were examined. Values represent mean ± s.e.m. (^#^ indicates *P* > 0.1; Bootstrap-test).

To assess whether STIM1 is required for axon guidance in vivo, we examined the midline axon guidance of commissural interneurons in the developing Xenopus spinal cord, which is known to require netrin-1 signaling [20,23]. Commissural interneuron axons in developing Xenopus embryos were specifically identified with the 3A10 monoclonal antibody [20]. In stage 25-26 embryos, 3A10-positive commissural axons extend toward and across the midline in a highly organized manner (Figure 7A and B). In contrast, a significant percentage of commissural axons derived from YFP-XSTIM1-DN injected embryos wondered around and failed to cross the midline with some even went out of the spinal cord (Figure 7C). For simplicity, we scored both types of guidance defects as the one not crossing and presented and quantified the percentage of crossed axons (Figure 7F). XSTIM1-DN markedly reduced the percentage of crossed axons to about 50% of the control groups (uninjected and GFP injection only) and the WT group. Similarly, over-expression of YFP-XSTIM-CA, but not YFP-XSTIM1-WT, also led to significant midline guidance defects (Figure 7D and F). On the other hand, none of these molecular manipulations significantly affected the numbers of commissural neurons in the developing Xenopus spinal cord (Figure 7G). Taken together, these results demonstrated that STIM1 is requried for the midline axon guidance of commissural interneurons in the developing Xenopus spinal cord in vivo.

## Discussion

Ca^2+^ is a key second messenger that mediates a wide range of neuronal activity and responses to extracellular stimuli [1]. Spatiotemporally-restricted Ca^2+^ signals can steer growth cones in responses to extracellular guidance cues and are thought to be generated from Ca^2+^ influx through the plasma membrane as well as Ca^2+^ release from internal store [1,18]. However, how these two events are coupled to sustain Ca^2+^ signaling during guidance responses remain unclear. SOCE is believed to be a part of the intracellular Ca^2+^ homeostasis machinery that maintains [Ca^2+^]_i_ for various neuronal functions, including optimal growth cone motility. The function of SOCE is well established in non-excitable cells and considered as a Ca^2+^ entry mechanism for refilling intracellular Ca^2+^ stores. The molecular components of SOCE include STIM, Orai, and TRP channels, which have also been identified and characterized mostly in non-excitable cells [2,3]. More recently, several studies support the presence and functional implication of SOCE in the nervous system [4-9], but the molecular composition and functional role of SOCE and STIM1 in neuronal growth cone guidance is not well established. Our data demonstrate that STIM1- and TRPC1-dependent SOCE not only operates in neuronal growth cones but also mediates filopodial Ca^2+^ transients and growth cone guidance both in vitro and vivo [20,30,31]. Our results are consistent with a recent report showing that STIM1 and Orai, two components of SOCE, are involved in growth cone responses to brain derived neurotrophic factor and Semaphorin-3a [32]. Importantly, our study has further identified that TRPC1 is also an essential component of SOCE and a major site of SOCE-mediated Ca^2+^ entry is in the filopodia, especially at the tip of the filopodia. Moreover, we have presented in vivo evidence that STIM1 is required for proper guidance of commissural axons in developing spinal cord, a classic example of netrin-1-dependent long-range growth cone guidance [20,30,31]. Together, the current study has clearly established a role for SOCE, involving STIM1 and TRPC1, in mediating filopodial Ca^2+^ entries underlying axonal growth cone guidance.

At this moment, the precise mechanism by which STIM1 and SOCE are involved in netrin-1-induced Ca^2+^ signaling for growth cone attraction remains unclear. Netrin-1 is known to elicit Ca^2+^ signals by Ca^2+^ influx through TRPC1 and Ca^2+^ release from the internal stores to induce growth cone attraction [1,18-20] but the underlying mechanism remains unclear. It has been shown that brain derived neurotrophic factor (BDNF) triggers Ca^2+^ release from internal stores through activation of the PLC-γ and IP_3_ pathway, which in turn induces SOCE through STIM1-TRPC activation [18,32-34]. Netrin-1 is considered to be in the same group of Ca^2+^-mediated guidance cues with BDNF. Given that PLC-γ and IP_3_-induced Ca^2+^ release are involved in growth cone extension and navigation [22,34-36], we propose that netrin-1 may initiate intracellular Ca^2+^ release through activation of netrin-1 receptor Deleted in Colorectal Cancer (DCC), PLC-γ and IP_3_ production, which further triggers store-depletion, STIM1 activation, and Ca^2+^ influx through TRPC1 for replenishing ER Ca^2+^. This notion is further supported by the findings that both netrin-1 and BDNF activate PLC-γ and Phosphatidylinositol 4,5-bisphosphate (PIP_2_) hydrolysis in neurite elongation [37,38]. Therefore, our results provide additional evidence for the conserved signaling pathways among Ca^2+^-mediated guidance cues and between netrin-1 and neurotrophins.

The role of TRPC channels as SOC has been controversial, but multiple lines of evidences support TRPC as a strong candidate component of store-operated Ca^2+^ channels. For example, TRPC1 has been shown to be bound and activated by STIM1 and contribute to SOCE in some cells [16,17,39-41]. We found that STIM1 interacts with TRPC1 in embryonic neural tissues (Additional file 2: Figure S2) and that TRPC1 knockdown inhibits STIM1-mediated SOCE within growth cones and filopodia (Figures 2D, 3D and 3E), suggesting that TRPC1 is an essential component of SOCE. As STIM1 is also required for netrin-1-induced Ca^2+^ elevation and growth cone attraction which was shown to be mediated by TRPC1, our data support a role for STIM1 in activating TRPC1. However, we cannot rule out the possibility that STIM1 may affect Ca^2+^ signaling and growth cone guidance by other mechanisms, such as its effects on cAMP signaling or ER remodeling [42,43]. Recent studies also showed biochemical assembly of STIM1-TRPCs-Orai complex and functional connections between TRPC channels and Orai1 [41,44,45]. STIM1-Orai1 co-localization in response to Ca^2+^ depletion was reported in neuronal growth cones [32]. Therefore, it is possible that Orai also plays a role in netrin-1 signaling and guidance.

It should be noted that Lck-GCaMP3 was successfully used in distinguishing the Ca^2+^ signals from membrane entry from internal release from the stores [25,46]. Our data with Lck-GCaMP3 showing the presence of filopodial Ca^2+^ transients and its potentiation by netrin-1 is consistent with the previous reports using Fluo-4 [14,15,26]. However, when compared with previous studies using Fluo-4, the incidence and frequency of filopodial Ca^2+^ transients observed in our study appear to be lower than those seen in the previous reports. The difference may be attributed to two possibilities. First, Lck-GCaMP3 detects Ca^2+^ entry events only at near-plasma membrane regions. However, fluo-4 could detect cytosol Ca^2+^ changes from other sources such as intracellular stores, which will likely be missed by Lck-GCaMP3. In this regard, Lck-GCaMP3 fluorescence Ca^2+^ signals may be better called “filopodial Ca^2+^ entries” rather than filopodial Ca^2+^ transients. Second, we did not count the Ca^2+^ transients propagated from the growth cone proper and only counted the Ca^2+^ entry events generated within the filopodium independently of Ca^2+^ transients from the growth cone proper. Therefore, our data do not contradict the previous work.

It is of interest to see that the initial site of filopodial Ca^2+^ entry is largely localized to the tip of filopodia. Filopodia are considered to be the sensory apparatus for growth cones as they extend farther distance to detect the environmental signals. Therefore, it makes sense to have the sensory molecules accumulated at the tip for signal transduction initiation. However, the lack of quality antibodies prevented us from convincingly detecting the localization of STIM1/TRPC1 and other SOCE components at the filopodial tip. On the other hand, it has been reported that several receptors such as integrins, TRPC1 and DCC [14,26,47] and signaling molecules such as Src, PAK, PKA [48,49] are enriched at the tip of filopodia along with many other cytoskeleton regulatory molecules [11,50,51]. Therefore, it is conceivable that STIM1 and TRPC1 could function at the filopodial tip as an effective way to sense the environment and initiate Ca^2+^ signaling during growth cone guidance.

We report fast, highly localized and periodic spontaneous filopodial Ca^2+^ entries initiated independently of growth cone Ca^2+^ transients, which was consistent with the previous reports of oscillatory pattern of spontaneous Ca^2+^ transients within growth cone and filopodia during axonal growth [14,15,52]. The critical role for TRPC1 in generation of filopodial Ca^2+^ entry and its potentiation by netrin-1 is also consistent with the previous reports [14,15,26]. A further unexpected result was that STIM1-DN mutant blocked not only the SOCE-induced filopodial Ca^2+^ entries that depend on STIM1 but also spontaneous and netrin-1-potentiated oscillatory filopodial Ca^2+^ entries, suggesting that STIM1-dependent SOCE mediates the generation and maintenance of filopodial Ca^2+^ entries. Thus, our visualization of oscillatory patterns of spontaneous filopodial Ca^2+^ entries and their inhibition by STIM1- or TRPC1-knockdown is the first demonstration of a role of STIM/TRPC1-dependent SOCE in regulating Ca^2+^ oscillation in neurons, which is consistent with previous findings in other cell types [53-55]. It is plausible that store Ca^2+^ release, transient drop in ER Ca^2+^, and Ca^2+^ entry through TRPC1 triggered by transient STIM1 activation may underlie the Ca^2+^ oscillations seen in growth cone filopodia. Thus, considering the functional correlation between the frequency of filopodial Ca^2+^ transients and growth cone outgrowth and turning [14,26], disruption of STIM1 or TRPC1 function is likely to result in the breakup of Ca^2+^ cycling for oscillations and subsequent attenuated frequency of filopodial Ca^2+^ entries, which may further cause the suppression of growth cone guidance in response to netrin-1.

## Conclusions

Our data demonstrate a role for STIM1/TRPC1-dependent SOCE in mediating oscillatory patterns of spontaneous and netrin-1-potentiated filopodial Ca^2+^ entries that underlie axonal growth cone guidance both in vitro and in vivo.

## Methods

### Molecular constructs

*Xenopus* STIM1 (XSTIM1) [GenBank: BC126011] was identified by BLAST searches of the GenBank database using human STIM1 cDNA sequence. The coding region of XSTIM1 gene was isolated by RT-PCR, sequenced and cloned into the pCS2 vector (gift of D. Turner, University of Michigan). The following constructs of XSTIM1 and its mutants were generated by site-directed mutagenesis (Strategene) or by PCR based on previous studies of mammalian STIM1 mutants [16]: YFP-XSTIM1-WT, YFP-XSTIM1-DN, YFP-XSTIM1-CA, and mCherry-XSTIM1-DN (Figure 2A). Different XSTIM constructs were *in vitro* transcribed with the mMESSAGE mMACHINE SP6 kit (Ambion). pN1-Lck-GCaMP3 plasmid was obtained from Addgene (plasmid #26974, [25]), cloned into pCS2 vector using BamHI and XbaI sites and in vitro transcribed with mMESSAGE mMACHINE SP6 kit (Ambion).

### Xenopus embryo injection and spinal neuron culture

Blastomere injections of mRNAs or morpholinos into early stages of *Xenopus* embryos and culturing of spinal neurons from these injected embryos were performed as previously described [20,23,29,56]. Briefly, fertilized embryos were injected at the two- or four-cell stage with mRNA (2-3 ng/embryo). A control morpholinos or morpholinos specific for X*enopus* TRPC1 (XTRPC1-MO) was previously described [20]. Uninjected or injected embryos at stage 22 were used for cultures of spinal neurons as previous described [20,23]. All the procedures involving *Xenopus* frogs and embryos were carried out in accordance to the NIH guideline for animal use and have been approved by the Institutional Animal Care and Use Committee (IACUC) of Emory University.

### RT-PCR and Whole-mount in situ hybridization of Xenopus embryos

Neural tube and notochord were isolated from the dorsal section of the stage 25-26 *Xenopus* embryos after dissection with microsurgical scissors and incubation with collagenase (type I, Sigma). Total RNA was prepared by using TRIzol Reagent (invitrogen) and treated with the RNase-free DNAse I (Roche) to remove genomic DNA. The extracted RNA was reverse transcribed by using M-MLV reverse transcriptase (Invitrogen) and random hexamers (Roche). PCR amplification was performed using Taq polymerase (Fermentas). The –RT lane is the negative control of the RT-PCR on neural tube tissue RNA in the absence of a reverese transcriptase. The PCR primers are as follows; XSTIM1-forward, 5′ CCAGAACCTTGGAAGAGGTG 3′, XSTIM1-reverse, 5′ GACTGAATGGTACCGGCTGT 3′; XODC-forward, 5′ CAGCTAGCTGTGGTGTGG 3′, XODC-rev, 5′ CAACATGGAAACTCACACC 3′. For whole-mount *in situ* hybridization, the digoxigenin (DIG)-UTP-labelled antisense RNA was used as previously described [23,57]. The C-terminal region of XSTIM1 corresponding to amino acid 192-668 was used for the specific anti-sense and sense probes. The labelled probe was detected with alkaline phosphatase-conjugated anti-DIG antibody (Fab fragments) and visualized with the BM purple AP substrate (Roche Applied Science). Selected embryos from whole-mount *in situ* hybridization were embedded in a sucrose and Tissue-Tek O.C.T medium, completely frozen and cross-sectioned at 40 μm with a cryostat (Leica CM1850).

### Immunocytochemistry

*Xenopus* spinal neuron cultures were fixed in 4% paraformaldehyde in a cacodylate buffer (0.1 M sodium cacodylate, 0.1 M sucrose, pH 7.4) for 30 minutes and permeabilized with Triton X-100 (0.1%) for 10 minutes. The cells were incubated with a rabbit polyclonal antibody against full length human STIM1 (MyBioScource) at a dilution of 1:50 after blocking with 5% goat serum and labelled with Alexa Fluor 546 goat anti-rabbit secondary (Invitrogen). Fluorescent imaging was captured on an inverted microscope (Nikon Eclipse Ti-E).

### Growth cone turning assay

Microscopic gradients of netrin-1 (5 μg/ml in the pipette) were produced as previously described [29,56,58,59]. *Xenopus* spinal neurons derived from injected blastomeres were identified under fluorescent microscope and used for turning assay at the room temperature 14 to 20 hrs after plating as previously described [20,23,29,56]. The culture was plated on glass coverslip without any coating. The turning angle was defined by the angle between the original direction of neurite extension and a straight line connecting the positions of the center of the growth cone at the onset and the end of the 30 min period. The rates of neurite extension were calculated based on the net neurite extension during the turning assay. Only those growth cones of isolated neurons with a net neurite extension > 5 μm over the 30-min period were included for analysis. Statistical significance was assessed using the Bootstrap-test.

### Ca^2+^ imaging of cultured Xenopus spinal neurons

Ca^2+^ imaging of cultured growth cones of *Xenopus* spinal neurons was performed as previously described [23,29,56]. Specifically, isolated *Xenopus* spinal neurons were cultured on glass coverslip without coating, loaded with Fluo-4 AM (2 μM, Molecular Probes) for 30 minutes, rinsed with the Modified Ringer used for growth cone turning assay, and imaged after bath-application of netrin-1 (10 ng/ml). For store-operated Ca^2+^ entry experiment, neurons were bathed in Ca^2+^ -free media with CPA (25 μM) to deplete intracellular Ca^2+^ stores, and imaged after re-addition of extracellular Ca^2+^ (1.5 mM). Growth cones expressing mCherry-XSTIM1-DN proteins were identified under fluorescent microscope and selected for further Ca^2+^ imaging. Imaging was carried out using a Zeiss 510 META system equipped with a 20X objective (NA 0.8). Excitation was at 488 nm by argon laser and the emitted fluorescence was collected at 500-560 nm. Fluorescence and bright-field images were simultaneously acquired at every 30 seconds with a frame scan. The mean fluorescence intensity of each time point was measured over a fixed circular region of interest that covers the entire growth cone and normalized to the average fluorescence intensity that was measured during a 2 minutes baseline period (prior to the netrin-1 application or addition of 1.5 mM Ca^2+^ solution).

For filopodial Ca^2+^ imaging, Lck-GCaMP3 mRNA was injected into early staged embryos without or with other mRNAs or morpholino. Spinal neurons were cultured on the glass coverslip coated with poly-D-lysine and laminin, which increases the number and length of filopodia [60], in serum-free culture medium. In our netrin-1-induced filopodial Ca^2+^ entries experiments, the spinal neurons were incubated in MR solution with the addition of cAMP analog Sp-cAMP (25 μM) to counterbalance the laminin’s effect of reducing cAMP levels in growth cones [61] and mimic the condition of our *in vitro* turning assay where laminin coating on the glass culture dish was omitted. Live cell imaging of Ca^2+^ transients was performed on an inverted microscope (Nikon Eclipse Ti-E) equipped with a 60X Apo TIRF objective (NA 1.49), and EMCCD camera (Photometrics) using NIS-Elements software (Nikon). Excitation was at 488 nm and the emitted fluorescence was collected at 520 nm and Lck-GCaMP3 fluorescence images were acquired at every 200 milliseconds. To determine several characteristics of filopodial Ca^2+^ entries, including the incidence, frequency and initiation sites of transients, the Kymographs (spatio-temporal map) were created from the images of the user-defined segmented line one pixel in width spanning the filopodium from the time-lapse movies with NIH ImageJ software. Grayscale values for this linear region of interest (ROI) for each frame of the time series were transformed into pseudocolored images to show time-dependent changes in intracellular Ca^2+^ concentration ([Ca^2+^]_i_) along the length of the ROI (y-axis) over time (x-axis).

### Immunoprecipitation and immunoblotting

For co-immunoprecipitation, protein lysates were prepared from the *Xenopus* brain explants including spinal cords dissected from the embryos at stage 26-28 using lysis buffer containing 1% Triton X-100, 150 mM NaCl, 10 mM Tris-Cl (pH 7.4), 1 mM EDTA, 1 mM EGTA, 0.5% Nonidet P-40, 0.2 mM Na-orthovanadate, and protease inhibitor cocktail. They were incubated with the appropriate antibody for 3-4 hours at 4°C, followed by incubation with protein A/G agarose beads (Pierce) for overnight at 4°C. Mouse anti-c-Myc monoclonal antibody (Roche Applied Science) and rabbit anti-GFP polyclonal antibody (Abcam) were used for immunoprecipitation and immunoblotting.

### Whole-mount immunocytochemistry

Embryos at stage 25-26 were fixed and processed for immunocytochemistry as previously described [20,23,62]. Monoclonal antibody 3A10, specific for commissural interneurons [23,63], was obtained from the Developmental Studies Hybridoma Bank at the University of Iowa and used at a dilution of 1:100. Secondary antibodies were used at a dilution of 1:250. Confocal images of sagittal views of embryos were taken with a Zeiss LSM 510 META system and Z-series reconstructions were processed with the Zeiss LSM image acquisition program as previously described [23]. Statistical significance was assessed using the Bootstrap-test.

## Competing interests

The authors declare that they have no competing interests.

## Authors’ contributions

SS designed and performed all the experiments, and wrote the manuscript. JQZ oversaw the project, designed some of the experiments, and revised the manuscript with SS. GLM provided guidance to the project and contributed to the writing of the manuscript. All authors read and approved the final manuscript.

## Supplementary Material

Additional file 1: Figure S1 Alignment of STIM1 amino acid sequences of *Xenopus laevis*, *Xenopus tropicalis* and human. Identical amino acid residues are highlighted in dark. Dashes indicate gaps inserted for maximal alignment score. The red box indicates the amino acid (D65, within the EF hand motif) that was mutated in the dominant negative form of XSTIM1. Click here for file

Additional file 2: Figure S2 Association of XSTIM1 with TRPC1. Shown are sample westernblots for co-immunoprecipitation of human TRPC1 (myc-hTRPC1-WT) and wild-type (YFP-XSTIM1-WT) or constitutively active STIM1 (YFP-XSTIM1-CA) expressed in *Xenopus* embryonic neural tissues. Click here for file

Additional file 3 **Movie 1.** Filopodial Ca^2+^ entries are generated by SOCE. A pseudocolored Lck-GCaMP3 fluorescent Ca^2+^ images of *Xenopus* neuronal growth cones were captured at 200 milliseconds intervals over 7 min period of the store-depletion and re-addition of extracellular Ca^2+^ as shown in Figure 4A. Click here for file

Additional file 4 **Movie 2.** SOCE-induced filopodial Ca^2+^ entries are abolished by inhibition of XSTIM1 with XSTIM1-DN overexpression. A pseudocolored Lck-GCaMP3 fluorescent Ca^2+^ images of *Xenopus* neuronal growth cones expressing XSTIM1-DN were captured at 200 milliseconds intervals over 7 min period of the store-depletion and re-addition of extracellular Ca^2+^. Click here for file

Additional file 5 **Movie 3.** SOCE-induced filopodial Ca^2+^ entries are attenuated by inhibition of XTRPC1 with XTRPC1-MO overexpression. A pseudocolored Lck-GCaMP3 fluorescent Ca^2+^ images of *Xenopus* neuronal growth cones expressing XTRPC1-MO were captured at 200 milliseconds intervals over 7 min period of the store-depletion and re-addition of extracellular Ca^2+^. Click here for file

Additional file 6 **Movie 4.** Oscillatory spontaneous filopodial Ca^2+^ entries. A pseudocolored Lck-GCaMP3 fluorescent Ca^2+^ images of *Xenopus* neuronal growth cones were captured at 200 milliseconds intervals over 7 min period in Modified Ringer solutions (1 mM extracellular Ca^2+^) as shown in Figure 5A. Click here for file

Additional file 7 **Movie 5.** Spontaneous filopodial Ca^2+^ entries are abolished by inhibition of XSTIM1 with XSTIM1-DN overexpression. A pseudocolored Lck-GCaMP3 fluorescent Ca^2+^ images of *Xenopus* neuronal growth cones expressing XSTIM1-DN were captured at 200 milliseconds intervals over 7 min period in Modified Ringer solutions. Click here for file

Additional file 8 **Movie 6.** Spontaneous filopodial Ca^2+^ entries are abolished by inhibition of XTRPC1 with XTRPC1-MO overexpression. A pseudocolored Lck-GCaMP3 fluorescent Ca^2+^ images of *Xenopus* neuronal growth cones expressing XTRPC1-MO were captured at 200 milliseconds intervals over 7 min period in Modified Ringer solutions. Click here for file

Additional file 9 **Movie 7.** Netrin-1-potentiated filopodial Ca^2+^ entries. A pseudocolored Lck-GCaMP3 fluorescent Ca^2+^ images of *Xenopus* neuronal growth cone were captured at 200 milliseconds intervals in Modified Ringer solution (1 mM extracellular Ca^2+^) with Sp-cAMP (25 μM) before and after bath application of netrin-1 (10 ng/ml) as shown in Figure 4D. Click here for file

Additional file 10 **Movie 8.** Dynamic translocation of STIM1 into neuronal filopodia in response to store-depletion. Time-lapse fluorescent images of *Xenopus* neuronal growth cone expressing YFP-XSTIM1 before (1 mM Ca^2+^) and after store Ca^2+^ depletion (0 mM Ca^2+^/CPA). Images were captured at 2 seconds intervals. Click here for file
