# Supplementary material for: A critical role for STIM1 in filopodial calcium entry and axon guidance
Source: Mol Brain. 2013 Dec 1;6:51. doi: 10.1186/1756-6606-6-51 (PMC3907062; doi:10.1186/1756-6606-6-51)
Supplement: Additional file 1: Figure S1 — Alignment of STIM1 amino acid sequences of Xenopus laevis, Xenopus tropicalis and human. Identical amino acid residues are highlighted in dark. Dashes indicate gaps inserted for maximal alignment score. The red box indicates the amino acid (D65, within the EF hand motif) that was mutated in the dominant negative form of XSTIM1. [file 1756-6606-6-51-S1.pdf]

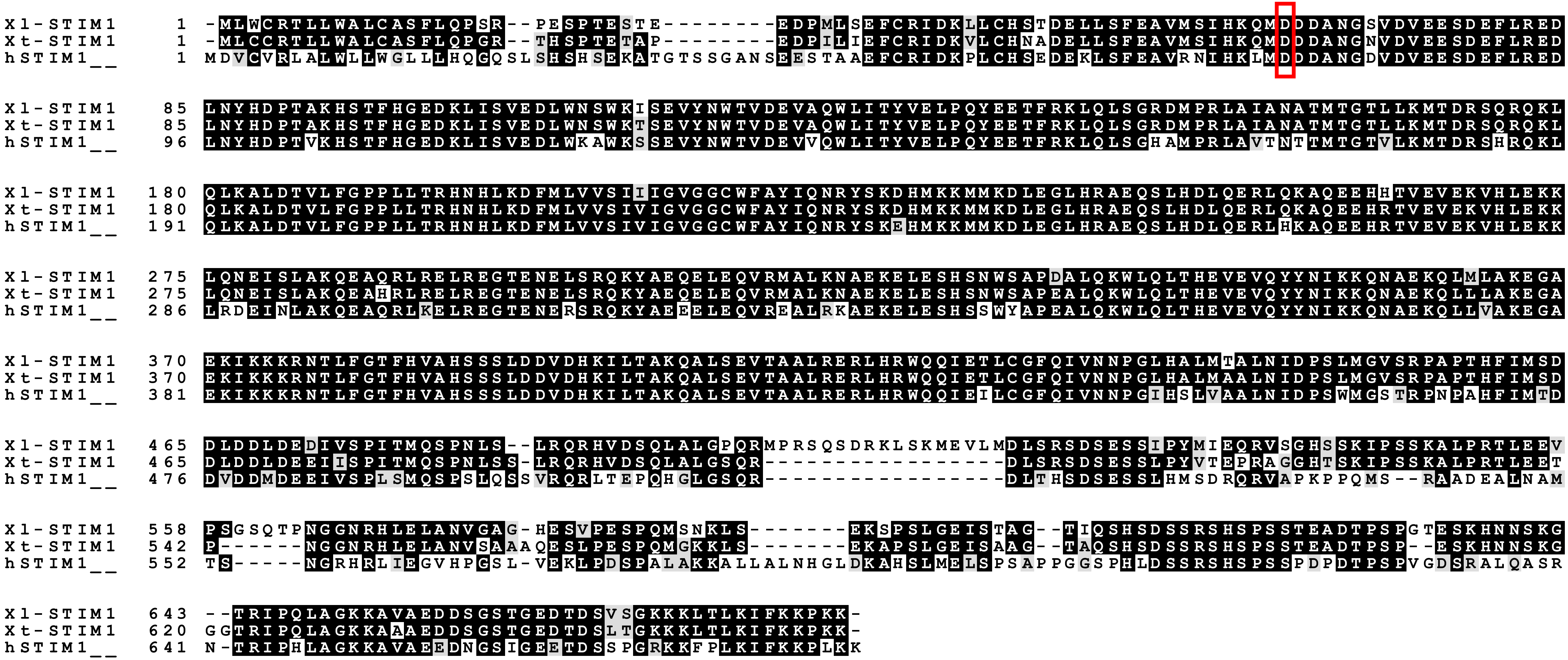

Additional file 1: Figure S1. (Shim et al.)
