# Supplementary figures and images for: A critical role for STIM1 in filopodial calcium entry and axon guidance
Source: Mol Brain. 2013 Dec 1;6:51. doi: 10.1186/1756-6606-6-51 (PMC3907062; doi:10.1186/1756-6606-6-51)

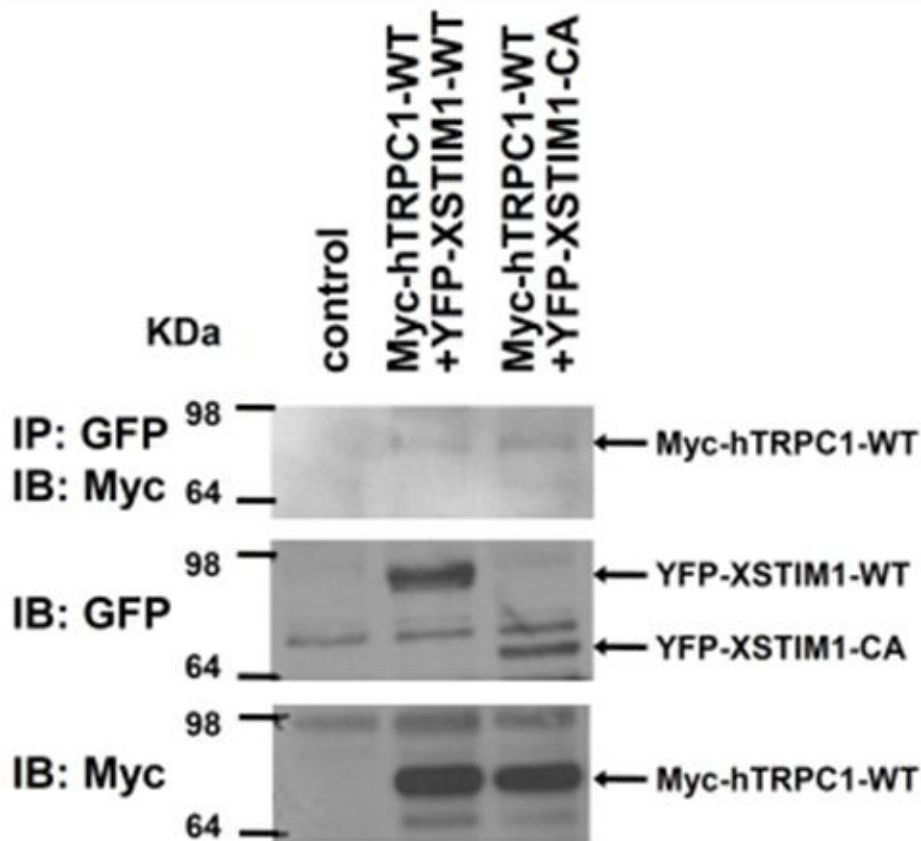

Additional file 2: Figure S2. (Shim et al.)

Supplement: Additional file 2: Figure S2 — Association of XSTIM1 with TRPC1. Shown are sample westernblots for co-immunoprecipitation of human TRPC1 (myc-hTRPC1-WT) and wild-type (YFP-XSTIM1-WT) or constitutively active STIM1 (YFP-XSTIM1-CA) expressed in Xenopus embryonic neural tissues. [file 1756-6606-6-51-S2.pdf]
